# Internal Degradation of 2.5D C/SiC Composites Under Continuous-Wave Laser Irradiation: Experiments and Phase-Selective Modelling

**DOI:** 10.3390/ma19163558

**Published:** 2026-08-21

**Authors:** Chuntong Liu, Renke Wang, Yuwei Lv, Yubin Shi

**Affiliations:** 1 Missile Engineering College, Rocket Force University of Engineering, Xi’an 710025, China; 2 State Key Laboratory of Laser Interaction with Matter, Northwest Institute of Nuclear Technology, Xi’an 710024, China

**Keywords:** 2.5D C/SiC, continuous-wave laser, internal degradation front, phase-selective degradation, multiscale heat transfer

## Abstract

**Highlights:**

- The internal degradation front extended 0.84–1.07 mm beneath the final recession surface.
- Phase-selective calculations related the measured front to local thermal and phase states without imposing a universal threshold.
- Surface recession alone underestimated subsurface damage in 2.5D C/SiC composites.
- Experimental-front mapping distinguished geometric removal from degradation within the retained material.

**Abstract:**

Surface recession can underestimate laser-induced damage in 2.5D C/SiC composites because thermochemical degradation extends beneath the visible pit. Infrared thermography and micro-CT data from six laser conditions (400–1600 W·cm^−2^, 3–12 s), together with SEM/EDS observations, were reanalysed using a layered phase-selective model tracking C, SiC and SiO_2_ evolution. For the four conditions with resolvable damage, the internal degradation front lay 0.84–1.07 mm below the recession surface. At 800 W·cm^−2^, the 0.02 mm difference between the 6 and 12 s front depths was below the 25 μm voxel size and within specimen uncertainty. The calibrated model matched rear-centre peak temperatures with a mean absolute percentage error of 4.37%, although larger transient discrepancies remained. Temperatures sampled at the measured front coordinates ranged from 2890 to 3080 K. Relative to 800 W·cm^−2^ for 12 s, the 1600 W·cm^−2^, 6 s condition caused greater near-surface SiC consumption and solid-mass loss, while the maximum retained SiO_2_ density decreased from approximately 360 to 180 kg·m^−3^. These results distinguish geometric recession from internal degradation and support experimental-front mapping and mechanistic interpretation. The mapped states are condition-specific and do not constitute an independently predicted front criterion.

## 1. Introduction

Carbon-fibre-reinforced silicon carbide (C/SiC) ceramic-matrix composites combine low density, high specific strength, thermal-shock tolerance and load-bearing capability at temperatures beyond the practical range of metallic structures. Peters et al. identified fibre-reinforced ceramics as an important part of the expanding materials space for hypersonic vehicles [1], while Naslain summarised how non-oxide fibres, matrices and interfaces can be designed to retain damage tolerance in severe thermal environments [2]. More recently, Canale and Citarella developed an iterative oxidation-damage framework for SiC/BN/SiC aero-engine structures [3], and Ammendola et al. reviewed the mechanical response and design opportunities of ultra-high-temperature ceramic-matrix composites [4]. These studies establish the structural value of C/SiC, but they also show that its performance depends on the coupled evolution of heat transfer, oxidation, cracking and constituent loss.

Laser irradiation provides a controllable route for reproducing extreme, spatially concentrated heat loads over short durations. Samant and Dahotre reviewed the absorption, melting, vaporisation and thermally induced fracture involved in laser machining of structural ceramics [5], and Tönshoff and Kappel showed that laser treatment can substantially modify ceramic surfaces through localised thermal processes [6]. For C/SiC specifically, Y. Wang et al. related macroscopic ablation morphology to oxidation and constituent removal [7], whereas Tong et al. identified distinct responses of the carbon reinforcement and SiC matrix under laser heating [8]. Pan et al. quantified the effects of laser parameters on surface morphology and ablation behaviour [9], and Yang et al. extended this comparison across a wider range of irradiation conditions [10]. Dang et al. examined the microstructural evolution of several carbon-fibre-reinforced SiC-based matrices and reported matrix-dependent products and transition-zone morphologies [11]. Wu et al. further showed that changing laser energy density alters recession, fibre exposure and product distribution in Cf/C–SiC composites [12]. Together, these experiments describe surface zonation in detail, but their assessment of damage remains based mainly on the final surface morphology.

The surrounding flow field changes both heat transfer and product removal. J. Wang et al. compared C/SiC ablation in static air and supersonic flow and found that airflow modified the pit morphology and recession rate [13]. Z. Wang, T. Ma and co-workers subsequently investigated transverse hypersonic airflow and demonstrated enhanced erosion and altered carbon-fibre morphology relative to static conditions [14]. The same group separated the thermal, mechanical and chemical contributions to the rapid ‘avalanche’ ablation observed in a hypersonic wind tunnel [15]. T. Ma et al. combined experiments and numerical analysis to examine the load-dependent ablation mechanisms of a Ti_3_SiC_2_-modified C/SiC composite under combined laser irradiation [16]. L. Wang et al. then coupled airflow experiments with an effective-thermal-conductivity description to predict temperature and ablation depth [17], and used the same framework to analyse the abrupt temperature rise observed during high-energy laser loading [18]. A related temperature-jump phenomenon was reported by Marschall et al. for ZrB_2_–SiC under plasmatron heating and was attributed to changes in surface chemistry and catalytic behaviour [19]. These results demonstrate that temperature histories and final recession are sensitive to atmosphere, product retention and property evolution, rather than to incident power alone.

Numerical studies have progressively incorporated these effects. Ge et al. developed an experiment-based model for laser-driven surface recession of C/C–SiC using evolving geometry and thermochemical removal [20]. J. Wang et al. introduced a multiscale description that distinguished the fibre and matrix responses and linked mesoscale evolution to macroscopic ablation under continuous-wave irradiation [21]. For architecture-dependent heat transfer, Zhao et al. coupled dynamic thermophysical-property evolution with multiscale heat transport in 2.5D C/SiC during air oxidation [22]. Sun et al. constructed microscale, void/matrix and mesoscale representative volume elements to calculate directional thermal conductivities for C/SiC composites with different preform architectures [23]. These approaches improve temperature and crater predictions, but they generally represent damage through a moving surface, an ablation temperature or a unified degradation variable.

The needled 2.5D architecture introduces an additional difficulty because continuous-fibre layers, chopped-fibre web layers, through-thickness needling fibres, matrix-rich regions and pores form different heat-transfer and gas-access pathways. Xu et al. showed that yarn dimensions influence the tensile and in-plane shear behaviour of needled textile ceramic composites, reflecting the structural role of the reinforcement architecture [24]. Zhao et al. further demonstrated that temperature-dependent anisotropy and multiscale transport govern thermal redistribution within 2.5D C/SiC [22]. In our previous continuous-wave laser experiments, micro-CT and cross-sectional SEM/EDS revealed a layered low-grey-value region whose boundary coincided with a marked change in the Si signal [25]. This boundary extended below the pit bottom, showing that substantial internal change occurred before complete geometric removal. It is termed the internal degradation front in the present study.

Composite oxidation studies provide a basis for interpreting this front. Lamouroux et al. experimentally showed that oxygen enters 2D woven C/SiC through coating cracks and consumes the internal carbon phase while silica develops on exposed SiC surfaces [26]. Their subsequent model coupled gas diffusion, carbon oxidation and silica growth and reproduced the measured mass evolution under different oxygen pressures and damage states [27]. Sullivan formulated a structural oxidation model that linked oxygen transport through cracks to recession of the carbon phase in C/SiC [28]. Eckel et al. quantified the oxidation kinetics of a continuous carbon phase embedded in a non-reactive matrix [29], and Opila and Serra demonstrated that carbon-fibre and C/SiC oxidation at reduced oxygen partial pressure can become strongly transport-controlled [30]. These studies explain why an internal reaction boundary may follow layer and pore pathways rather than the final external contour.

The SiC matrix introduces a second set of competing processes. Jacobson et al. described active oxidation as the formation of volatile SiO when a stable SiO_2_ layer cannot be maintained [31]. Narushima et al. measured rapid high-temperature oxidation of chemical-vapour-deposited SiC in wet oxygen [32], while Opila showed that water-vapour pressure strongly changes SiC oxidation kinetics [33]. Balat-Pichelin et al. mapped passive-to-active transitions for ceramic-matrix materials under re-entry-relevant conditions [34]. Chen et al. compared static and dynamic high-temperature oxidation and showed that gas flow changes the oxide morphology and reaction regime [35]. Under laser irradiation, Wei et al. directly related SiC/SiC surface morphology and component evolution to active/passive oxidation and product loss [36]. Accordingly, retained SiO_2_ is not a monotonic damage indicator: it is the net result of formation, transport and high-temperature volatilisation.

Despite this progress, most laser-ablation studies validate models against surface temperature, mass loss or crater geometry. These observables identify material that has already been heated or removed but do not directly describe the partially degraded volume that remains below the pit. Conversely, oxidation models resolve constituent consumption and oxygen transport but are rarely connected to an experimentally observed internal front under transient laser loading. A model that links the measured subsurface boundary to local temperature, remaining SiC, total solid mass and SiO_2_ retention is therefore needed to distinguish reaction progress from geometric recession.

Building on the observations reported in Ref. [25], this study combines reanalysed infrared thermography, surface morphology, micro-CT and SEM/EDS results from six continuous-wave laser conditions with a three-dimensional layered heat-transfer and phase-selective degradation model. Rear-centre temperature is used to evaluate the bulk through-thickness thermal response, while the previously identified inner boundary of the surface transition zone provides a secondary reference for the radial temperature field. After thermal calibration, the model tracks the evolution of C, SiC, SiO_2_ and total solid mass without using the experimental internal-front depth to readjust the model parameters. The experimentally measured front is subsequently mapped onto the calculated temperature and phase-state fields to characterise its associated material state and to interpret how thermal loading, constituent consumption and SiO_2_ formation–loss competition contribute to the separation between surface recession and internal degradation.

## 2. Materials and Experimental Methods

### 2.1. Material and Specimen Preparation

The material was a 2.5D needled C/SiC composite comprising alternating 0° and 90° continuous-fibre layers and chopped-fibre web layers connected by through-thickness needling fibres. The reinforcement consisted of T700 12K PAN-based carbon fibres and the matrix was SiC. The material architecture, fabrication route and preform schematic were reported in Ref. [25] and are not reproduced here.

The as-received material was machined into 40 mm × 40 mm × 3 mm specimens. The irradiated and opposite faces are hereafter termed the front and rear faces, respectively, and the through-thickness direction is denoted by z. Surface-recession and internal-front depths were measured from the original front surface in an unaffected region.

### 2.2. Laser Irradiation Tests

Laser tests were conducted in quiescent air using the continuous-wave laser and infrared-thermography arrangement detailed in Ref. [25]. Briefly, a 1070 nm fibre laser was collimated and shaped before normal incidence on the vertically mounted specimen. The diameter enclosing 80% of the beam energy was 21 mm, and the beam and specimen centres were aligned. The previously published apparatus schematic is not reproduced here.

Three power densities (400, 800 and 1600 W·cm^−2^) and two irradiation durations at each level produced six conditions (Table 1). Conditions are denoted as power density–duration; for example, 800–12 indicates 800 W·cm^−2^ for 12 s. Tests at 400 W·cm^−2^ produced primarily surface thermal modification, whereas 800 and 1600 W·cm^−2^ produced measurable surface recession and internal low-density regions.

The original tests and surface zonation were reported previously [25]. Here, the temperature, micro-CT and EDS data were reprocessed for the present modelling objectives: rear-centre temperature was used to evaluate the bulk thermal response, surface morphology was compared with near-surface phase distributions, and micro-CT/EDS identified the internal degradation front and its compositional characteristics.

### 2.3. Temperature Measurement

Two FLIR infrared cameras simultaneously recorded the front- and rear-face responses at 25 Hz and 640 × 480 pixels. The emissivity was set to 0.95, and both systems were operated over a calibrated high-temperature range of 500–3000 °C (773.15–3273.15 K). The acquisition software continued to return numerical outputs before a measured region entered this range. Such sub-range outputs do not represent calibrated physical temperatures and were excluded from thermal calibration and quantitative error evaluation. The numerical model was independently initialised at the ambient temperature of 293.15 K.

Rear-centre temperature was used to quantify the bulk through-thickness response. Front-face temperature was extracted along a radial line through the beam centre, and transition-zone boundaries identified from the post-test morphology were registered onto the temperature field immediately before laser shut-off. Rear-face temperature served as the principal quantitative metric; the inner transition-boundary temperature and radius reported previously [25] were used to evaluate the radial field in Section 4.2. Front-centre measurements were interpreted with consideration of emissivity, spatial resolution and registration uncertainty [37,38].

### 2.4. Micro-CT and SEM/EDS Characterisation

Post-irradiation internal structures were characterised by micro-CT [39,40] using a Bruker SkyScan 1275 system (Bruker Corporation, Billerica, MA, USA) at 70 kV and 80 μA, with a 100 ms exposure, a 1 mm Al filter and a 0.5° rotation step. The reconstructed voxel size was 25 μm. All specimens were reconstructed with identical settings and a reference intensity of 58,000 to preserve grey-value comparability.

The original specimen surface was defined using the intact peripheral region outside the irradiated zone and was used as the common depth datum. Measurements were performed on the central XZ and YZ sections and on four parallel sections located 0.10 mm on either side of the two central planes, giving six fixed sections for each specimen. Surface recession was measured from the original-surface datum to the continuous crater surface, and internal-front depth was measured from the same datum to the internal grey-value transition. The six section-level measurements were averaged to obtain one specimen-level value. Two independently irradiated specimens were analysed for each condition, and the reported standard deviation was calculated from the two specimen-level means.

Representative specimens were sectioned through the beam centre to relate the CT transition to composition. After grinding and polishing, the sections were examined using a Zeiss Sigma 300 scanning electron microscope (Carl Zeiss Microscopy GmbH, Jena, Germany), and C, Si and O distributions were mapped by EDS. Accelerating voltages of 3–10 kV were selected according to magnification and mapping requirements, with comparable imaging and mapping conditions applied to modified and relatively intact regions.

## 3. Numerical Methodology

### 3.1. Multiscale Geometry and Heat Transfer

A layered effective heat-transfer framework was constructed using two scales and three representative volume elements (RVEs). The microscale fibre-bundle RVE explicitly separated carbon fibres from the SiC/PyC matrix and normalised the solid composition of a fibre layer. Hierarchical pores were excluded at this scale to avoid double counting. Steady temperature differences imposed along the three material axes yielded the anisotropic effective properties of the unidirectional bundle.

**Figure 1:**
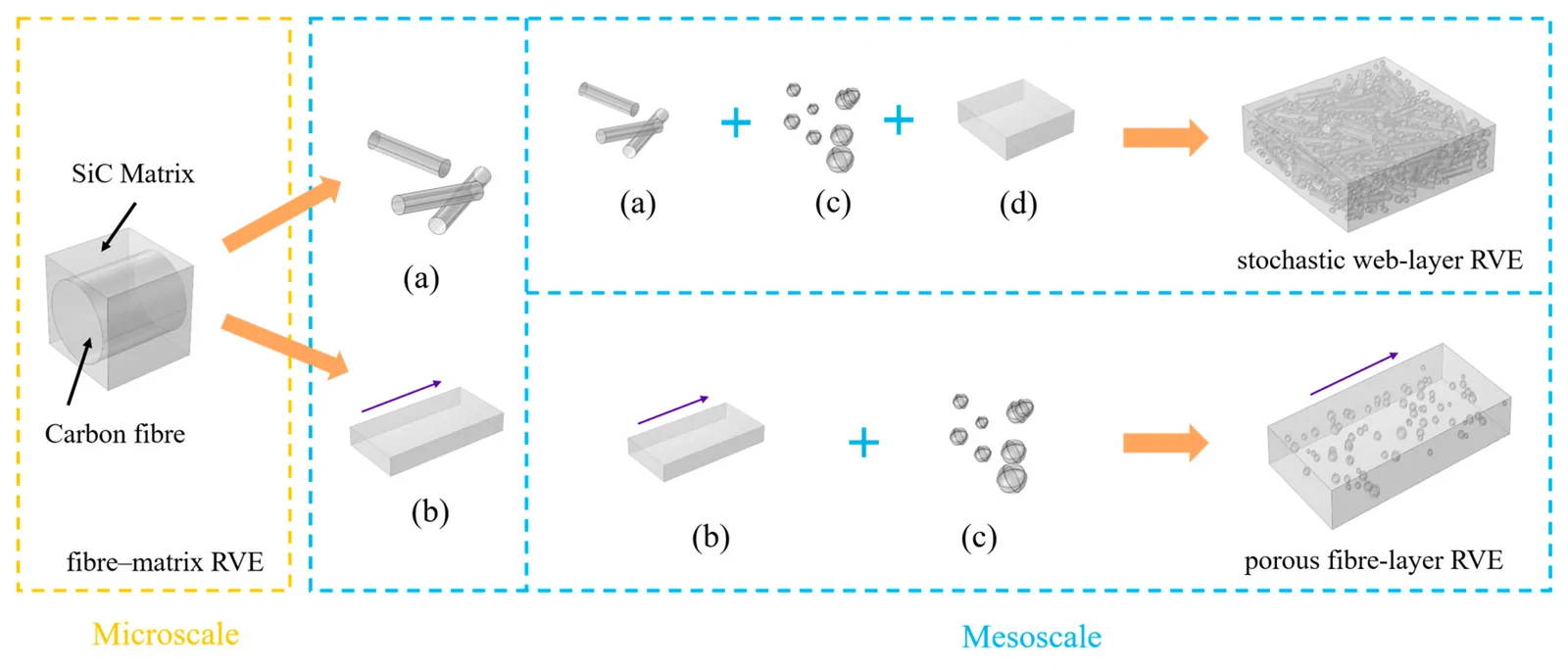
Multiscale construction of the RVEs for the 2.5D C/SiC composite. The effective thermophysical properties of the fibre bundles were obtained by homogenising the microscale fibre–matrix RVE and subsequently transferred to the mesoscale stochastic web-layer and porous fibre-layer RVEs. Here, (**a**) short fibre bundles in the web layer; (**b**) continuous-fibre bundles in the fibre layer; (**c**) pores; and (**d**) SiC matrix.

At the mesoscale, separate fibre-layer and web-layer RVEs were developed. The fibre-layer RVE treated the homogenised bundles and surrounding matrix as anisotropic continua and explicitly represented the larger pores rather than individual fibres. In the web-layer RVE, finite cylinders with random in-plane-biased orientations represented chopped bundles, while spherical pores were introduced to avoid overlap and facilitate robust meshing; the remaining volume was assigned to the effective matrix. This procedure established the microscale-bundle–mesoscale-layer–macroscale-laminate homogenisation path shown in Figure 1, consistent with multiscale descriptions of architecture-dependent heat transfer and porosity in C/SiC composites [22,23].

The RVE hierarchy provides directional, temperature-dependent thermophysical inputs for the macroscale layered model; it is not used to interpret local failure or reaction fields. Constituent allocation, geometry-generation settings, temperature-dependent inputs, final mesh descriptors and heat-flux checks are provided in the Supplementary Materials. A single adopted stochastic web-layer realisation was used. Because no multiple-realisation or RVE-size convergence series was retained, statistical representativeness is supported only by the prescribed phase fractions, directional plausibility and analytical-bound checks and is explicitly treated as a limitation. The influence of through-thickness transport uncertainty is assessed at the macroscale.

The macroscale dimensions match the specimen (40 mm × 40 mm × 3 mm). The irradiated face is the front face, and the through-thickness coordinate is z; the rear face is at z=0 and the original front surface at z=H, where H=3 mm. Depth from the original front surface is defined as

(1) d=H−z

A half model was used by exploiting specimen and beam symmetry. The thickness was divided into ten 0.3 mm layers, with continuous-fibre and web layers arranged alternately and successive continuous-fibre layers oriented at 0° and 90°. Needling fibres were not explicitly discretised. A through-thickness conductivity multiplier, $f_{z}$, was retained to examine possible unresolved contributions from the needling architecture and interlayer effects. Its final value was 1.00; therefore, no additional empirical scaling of the homogenised through-thickness conductivity was applied in the reported model. Temperature and heat flux were assumed to be continuous across the layer interfaces, with no additional empirical contact resistance.

The transient energy-conservation equation for layer l is

(2) $$\rho_{l,0}C_{p,l}\left(T\right)\frac{\partial T}{\partial t}=\nabla \cdot \left[K_{l}\left(T\right)\nabla T\right]+Q_{\mathrm{rxn}}$$

Here, subscript l denotes the structural-layer type; $\rho_{l,0}$, $C_{p,l}$ and $K_{l}$ are its initial effective density, specific heat capacity and thermal-conductivity tensor, respectively, and $Q_{\mathrm{rxn}}$ is the volumetric reaction-heat source. In the material coordinates, the conductivity tensor is

(3) $$K_{l}\left(T\right)=\mathrm{diag}\left[k_{x,l}\left(T\right),\;k_{y,l}\left(T\right),\;f_{z}k_{z,l}\left(T\right)\right]$$

The 0° and 90° continuous-fibre layers are represented by interchanging the principal *k_x_* and *k_y_* directions, whereas the web layer has independent effective properties in three directions. Properties are interpolated over 293.15–3500 K and held at endpoint values outside this range. The energy equation retains the initial effective *ρ*_*l*,0_, *C_p,l_* and *K_l_*, while the phase-derived local solid mass density acts as the degradation variable. Reaction heat *Q*_rxn_ contributes to the temperature field, but degraded-state thermophysical properties are not updated.

The continuous-wave laser is represented by an axisymmetric Gaussian heat flux. The experimental power density $I_{\mathrm{avg}}$ is the mean within the circle containing 80% of the beam energy; the incident power within this circle is

(4) $$P_{L}=I_{\mathrm{avg}}\pi R_{80}^{2},\;\;R_{80}=10.5\;mm$$

The Gaussian-beam $1/e^{2}$ radius $w_{0}$ satisfies

(5) $$1-\exp\left(-\frac{2R_{80}^{2}}{w_{0}^{2}}\right)=0.8,\;\;w_{0}=11.7049\;mm$$

The absorbed front-face heat flux is written in normalised form as

(6) $$q_{L}\left(r,t\right)=\eta_{\mathrm{abs}}\frac{2P_{L}}{\pi w_{0}^{2}\left[1-\exp\left(-2R_{80}^{2}/w_{0}^{2}\right)\right]}\exp\left(-\frac{2r^{2}}{w_{0}^{2}}\right)S_{L}\left(t\right)$$

Here, $\eta_{\mathrm{abs}}$ is the effective absorptivity, $S_{L}\left(t\right)$ is the smoothed laser switching function, and r is the radial distance from the beam centre. The front-face boundary condition is

(7) $$-n\cdot \left(K\nabla T\right)=q_{L}-h_{\mathrm{top}}\left(T-T_{\infty }\right)-\varepsilon \sigma \left(T^{4}-T_{\infty }^{4}\right)$$

The side and rear faces satisfy

(8) $$-n\cdot \left(K\nabla T\right)=-h_{j}\left(T-T_{\infty }\right)-\varepsilon \sigma \left(T^{4}-T_{\infty }^{4}\right),\;j\in \{side,back\}$$

The principal geometric and heat-transfer parameters are summarised in Table 2. Initial phase fractions, effective densities and temperature-dependent properties of the individual layers are provided in the Supplementary Materials.

### 3.2. Phase-Selective Degradation Formulation

#### 3.2.1. Reaction Pathways and Rate Formulation

Solid-mass evolution under continuous-wave irradiation includes oxidation of C, passive and active oxidation of SiC, high-temperature loss of Si-containing species, and SiO_2_ formation and loss. Composite-scale oxygen transport and carbon consumption follow established C/SiC oxidation descriptions [26,27,28,29,30], while the SiC and SiO_2_ pathways are based on passive/active oxidation and high-temperature product evolution [31,32,33,34,35,36]. Gaseous CO, CO_2_ and SiO are represented by equivalent solid consumption or production rates. The reaction pathways are

C(s) + ν_C_ O_2_(g) → CO_x_(g) SiC(s) + 3/2 O_2_(g) → SiO_2_(s/L) + CO(g) SiC(s) + O_2_(g) → SiO(g) + CO(g) SiC(s) → Si(g) + C(s) SiO_2_(s/L) → SiO(g) + 1/2 O_2_(g) (9)

In the carbon-oxidation pathway in Equation (9), *x* = 1 or 2 denotes CO or CO_2_, respectively, and *ν_C_* is the corresponding stoichiometric oxygen coefficient (1/2 for CO and 1 for CO_2_). Equation (9) defines the evolution directions of the solid-phase states. The equivalent local rate coefficient for reaction *j* is

(10) $$r_{j}=A_{j}\exp\left(-\frac{E_{j}}{R_{g}T}\right)F_{j}\left(T\right)\;a_{O_{2}}^{\chi_{j}}P_{j}\left(Y_{SiO_{2}}\right)$$

(11) $$F_{j}\left(T\right)=\frac{1}{2}\left[1+\tanh\left(\frac{T-T_{j,\mathrm{on}}}{\Delta T_{j}}\right)\right],\;\;P_{j}\left(Y_{SiO_{2}}\right)=\exp\left(-\beta_{j}Y_{SiO_{2}}\right)$$

Here, $A_{j}$ and $E_{j}$ are the effective pre-exponential factor and activation energy, respectively; $R_{g}$ is the universal gas constant; $T_{j,\mathrm{on}}$ and $\Delta T_{j}$ define the onset temperature and transition width; and $P_{j}$ represents inhibition by retained SiO_2_. The parameters were selected from literature-consistent ranges and bounded numerical trials and should therefore be interpreted as effective model inputs rather than uniquely identified elementary kinetic constants. Oxygen-consuming rates use the positive normalised oxygen-availability field, $a_{O_{2}}^{+}=\max\left(a_{O_{2}},0\right)$, whereas oxygen-independent high-temperature loss pathways are not multiplied by the oxygen-availability term. For concise presentation, the numerical subrates implemented in the model are grouped into the five physical reaction pathways in Equation (9); the complete subrate definitions and parameter settings are provided in Tables S8 and S9.

The normalised oxygen-availability field was solved throughout the ten solid domains according to

(12) $$\phi_{\mathrm{eff}}\frac{\partial a_{O_{2}}}{\partial t}-\nabla \;\cdot \;\left(D_{O_{2},\mathrm{eff}}\nabla a_{O_{2}}\right)=-\frac{R_{O_{2}}}{c_{O_{2},\infty }},\;\;D_{O_{2},\mathrm{eff}}=D_{\mathrm{air}}\frac{\phi_{\mathrm{eff}}^{1.5}}{\tau_{\mathrm{conn}}}$$

The initial value and the value prescribed on all exposed surfaces were $a_{O_{2}}=1$. A zero-normal-flux condition was applied to the symmetry boundary, and oxygen availability and normal diffusive flux were continuous across the internal layer interfaces. Because $a_{O_{2}}$ is normalised, it represents effective oxygen availability rather than an absolute concentration or partial pressure.

The stoichiometric oxygen demand comprises carbon oxidation and passive and active SiC oxidation. To prevent the oxygen-consuming rates from exceeding the locally available oxygen inventory, the provisional demand was compared with the local stock-limited rate

(13) $$R_{O_{2},\mathrm{stock}}=\frac{c_{O_{2},\infty }\phi_{\mathrm{eff}}a_{O_{2}}^{+}}{\tau_{O_{2},\mathrm{guard}}},\;\;\tau_{O_{2},\mathrm{guard}}=0.002s$$

A smooth limiter was applied only to the numerical subrates that consume oxygen. High-temperature SiC decomposition, carbon sublimation and SiO_2_ loss remained oxygen-independent in the present implementation. This continuum treatment represents internal oxygen availability and consumption but does not resolve the external gas boundary layer or crack-scale transport pathways.

#### 3.2.2. Phase-State and Density Evolution

The model tracks the C, SiC and SiO_2_ solid states $Y_{C}$, $Y_{SiC}$ and $Y_{SiO_{2}}$, respectively. $Y_{C}$ and $Y_{SiC}$ are the remaining fractions of their initial solid contributions, whereas $Y_{SiO_{2}}$ represents SiO_2_ formed by SiC oxidation and subsequently available for loss. For the pathways in Equation (9), their evolution is summarised as

(14) $$\frac{\partial Y_{C}}{\partial t}=-r_{C,ox}Y_{C}$$

(15) $$\frac{\partial Y_{SiC}}{\partial t}=-\left(r_{pass}+r_{act}+r_{dec}\right)Y_{SiC}$$

(16) $$\frac{\partial Y_{SiO_{2}}}{\partial t}=\gamma_{pass}r_{pass}Y_{SiC}-r_{SiO_{2},vol}Y_{SiO_{2}}$$

Here, $\gamma_{pass}$ is the effective conversion factor between SiC consumption by passive oxidation and SiO_2_ formation. The rate terms *r*_C,ox_, *r*_pass_, *r*_act_, *r*_dec_ and *r*_SiO2,vol_ correspond to carbon oxidation, passive SiC oxidation, active SiC oxidation, SiC decomposition and SiO_2_ volatilisation in Equation (9), respectively. Continuous bounds are imposed on all state variables to prevent non-physical negative values and numerical overshoot. The current solid mass density is

(17) $$\rho_{tot}=\rho_{C}^{*}V_{C,0}Y_{C}+\rho_{SiC}^{*}V_{SiC,0}Y_{SiC}+\rho_{SiO_{2}}^{*}V_{SiO_{2},ref}Y_{SiO_{2}}$$

(18) $$\rho_{rel}\left(x,t\right)=\frac{\rho_{tot}\left(x,t\right)}{\rho_{tot}\left(x,0\right)},\;\;\rho_{SiO_{2},cur}=\rho_{SiO_{2}}^{*}V_{SiO_{2},ref}Y_{SiO_{2}}$$

Here, ρ_i_^*^ and *V*_*i*,0_ are the intrinsic density and initial volume fraction of phase *i*, respectively, and *V*_SiO2,ref_ is the reference volume fraction for the SiO_2_ state. Because SiO_2_ may form by SiC oxidation, *ρ*_rel_ represents total solid mass relative to its initial value and may exceed unity during product formation. It is used to compare solid-mass changes across locations and conditions.

The reaction heat is calculated from the progress of each process and added to Equation (2):

(19) $$Q_{rxn}=-\sum_{j}\Delta H_{j}{\dot{\xi }}_{j}$$

Here, $\Delta H_{j}$ and ${\dot{\xi }}_{j}$ are the reaction enthalpy and volumetric reaction-progress rate, respectively. Reaction heat affects the temperature field, but the density changes in Equations (17) and (18) do not update the effective thermophysical properties.

#### 3.2.3. Model Outputs and Experimental Mapping

The fixed-grid model describes internal degradation through phase-state evolution without moving the free surface. Experimental recession quantifies macroscopic geometric removal, whereas simulated temperature, normalised oxygen availability *a*_O2_, remaining SiC state *Y*_SiC_, relative solid mass density *ρ*_rel_ and current SiO_2_ mass density *ρ*_SiO2,cur_ describe the internal thermochemical state. Together, these outputs relate the geometric-removal boundary to the internal degradation boundary.

To establish a testable link between the internal front and simulated thermal state, the experimental front depth $d_{f}^{exp}$ is first determined from micro-CT/EDS as described in Section 2. The simulated temperature is then sampled at the same centreline position and analysis time $t_{f}$:

(20) $$T_{f,map}=T\left(r=0,\;d=d_{f}^{exp},\;t=t_{f}\right)$$

*T*_f,map_ is the temperature sampled at the experimental-front position and links front depth to local thermal state. No marker is assigned where a resolvable front was not observed. Centreline *ρ*_rel_ and *Y*_SiC_ profiles compare through-thickness phase evolution, near-surface *ρ*_SiO2,cur_ characterises product-retention zones, and *a*_O2_ serves as an internal constraint on oxygen-consuming reactions.

#### 3.2.4. Numerical Implementation

The model was solved in COMSOL Multiphysics 6.4 using a transient finite-element formulation. The heat Equation (2), normalised oxygen diffusion–reaction equation, phase-state Equations (14)–(16), reaction heat source, and oxygen-inventory constraint were solved in a coupled manner within the same three-dimensional layered geometry. The mesh was locally refined in the beam-affected region and through the specimen thickness to resolve the steep front-face thermal gradient and the alternating 0.3 mm layers, while coarser elements were used in remote in-plane regions. Continuous smoothing was applied to laser switching, thermal activation, state-variable bounds and the oxygen-inventory constraint to reduce nonlinear oscillations near numerical thresholds.

Thermal calibration and phase-state analysis were performed sequentially. A preliminary heat-transfer-only joint grid scan considered η_abs = 0.75–0.90 and a through-thickness conductivity multiplier of 0.70–1.00 using the 400–12, 800–6 and 800–12 datasets; a weighted score emphasised rear-centre peak temperature and used the 800 W·cm^−2^ transition-zone references as secondary constraints. The later fully coupled six-condition model used the final audited values η_abs = 0.72 and f_z,eff = 1.00. The retained model file does not preserve a complete optimisation record for the subsequent change to η_abs = 0.72; therefore, no claim of a unique optimum is made. Thermal inputs were fixed before phase-state interpretation, and the CT/EDS front was not used to readjust them. Because temperature observations informed parameter selection, the comparisons below quantify cross-condition calibration performance rather than independent thermal validation.

## 4. Results and Discussion

### 4.1. Experimental Damage Characteristics

**Figure 2:**
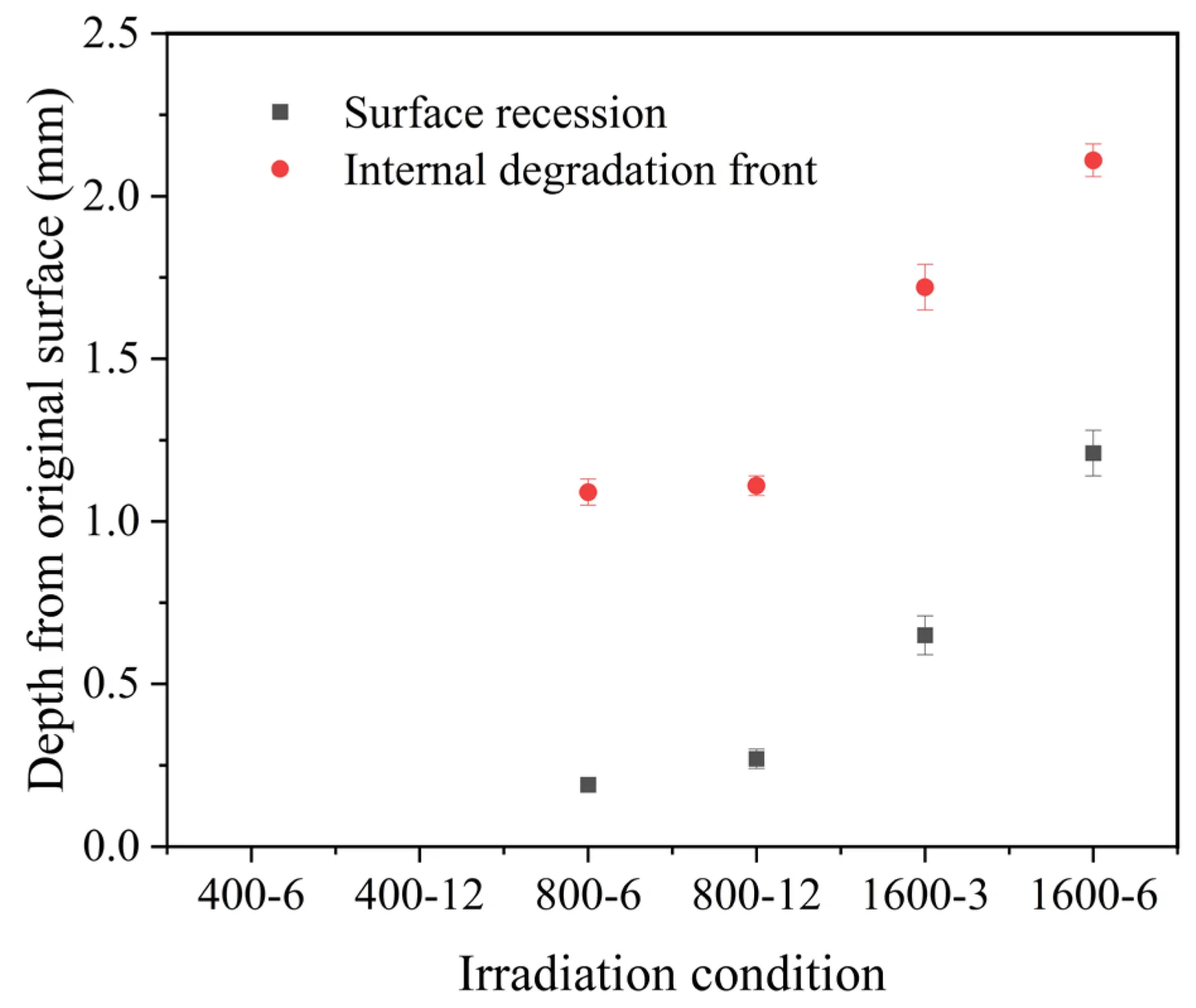
Reanalysed surface-recession and internal-degradation-front depths. Symbols show mean ± standard deviation for n = 2 independently irradiated specimens; each specimen value is the mean of six fixed CT sections. N.R., not resolvable at the 25 μm reconstructed voxel size.

The surface morphologies used to define the affected zones were previously reported in Ref. [25] and are not reproduced here. The present study instead provides a revised quantitative analysis of the surface-recession and internal-front depths using the fixed six-section CT protocol described in Section 2.4 (Table 3 and Figure 2). Neither resolvable surface recession nor a continuous internal degradation front was identified under the 400–6 and 400–12 conditions at the present CT resolution. Under the 800 W·cm^−2^ conditions, the mean internal-front depths after 6 and 12 s differed by only 0.02 mm. Because this difference was smaller than the reconstructed voxel size and lay within specimen-to-specimen uncertainty, it is not interpreted as resolved front advancement.

For all four conditions with resolvable damage, the internal-front depth exceeded the corresponding recession depth by 0.84–1.07 mm. The macroscopic pit represents the region from which material had been geometrically removed, whereas the deeper CT interface identifies a subsurface region that retained its overall geometry but exhibited a detectable change in reconstructed grey value. This contrast is consistent with internal constituent loss and pore development, although local phase fractions and interfacial weakening cannot be quantified directly from the present CT data. At 800 W·cm^−2^, no resolvable increase in internal-front depth was identified between 6 and 12 s. In contrast, increasing the irradiation duration from 3 to 6 s at 1600 W·cm^−2^ increased the measured recession and internal-front depths by 0.56 and 0.39 mm, respectively.

**Figure 3:**
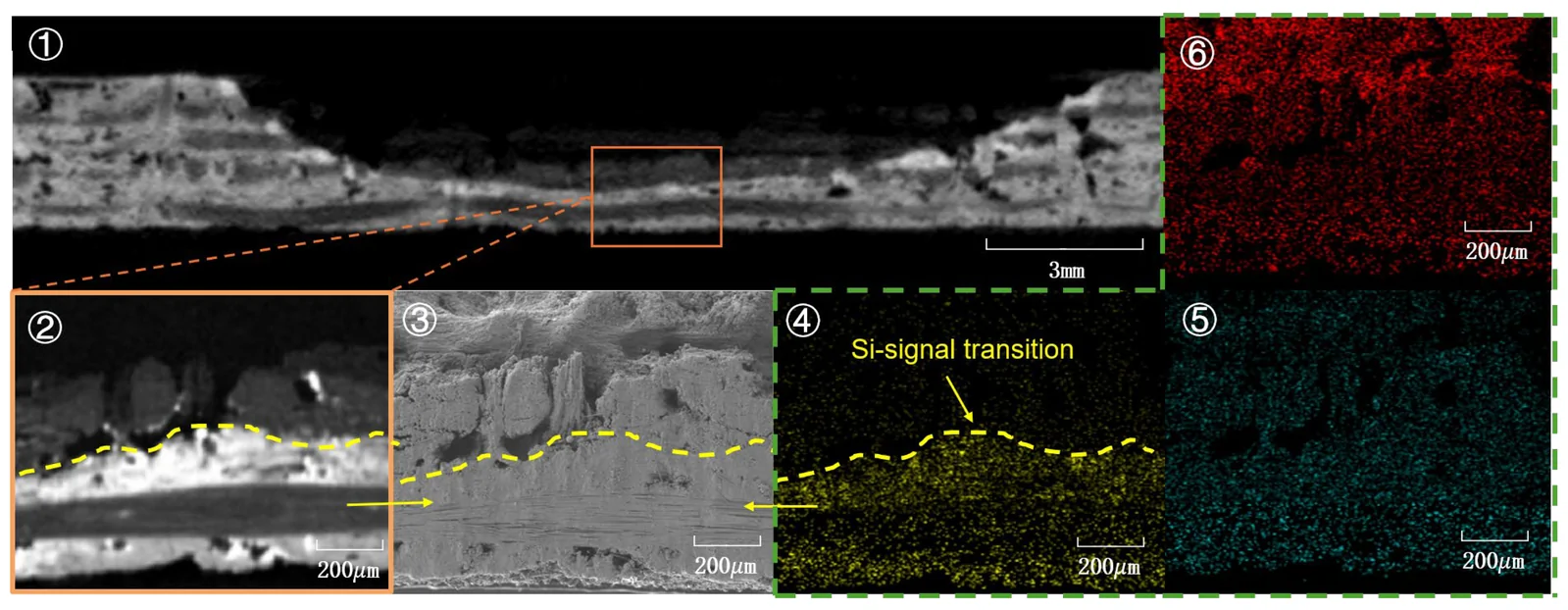
Cross-sectional characterisation of the internal degradation interface in the 2.5D C/SiC composite after irradiation at 1600 W·cm^−2^ for 6 s: (**1**) overall longitudinal micro-CT section; (**2**) enlarged CT view of the selected region; (**3**) corresponding SEM morphology; and (**4**–**6**) EDS maps of Si, O and C, respectively. The orange box and connecting lines indicate the selected region. Experimental panels adapted from Ref. [25] under the CC BY 4.0 licence and used here as qualitative context for model-state interpretation. The CT and EDS observations are not treated as quantitative measurements of local C, SiC or SiO_2_ phase fractions.

Cross-sectional micro-CT, SEM and EDS observations previously reported in Ref. [25] provide experimental context for the 1600–6 internal interface (Figure 3). They are re-used here only to locate the measured boundary relative to the newly calculated phase-state fields. Micro-CT shows a continuous grey-value transition below the pit, while SEM and EDS support structural and elemental differences across the selected region. The Si-signal transition is qualitatively consistent with the CT interface, but these observations do not provide quantitative local C, SiC or SiO_2_ phase fractions. Accordingly, SiC depletion, carbon consumption and pore development are treated as model-based interpretations rather than mechanisms quantified directly by CT or EDS.

### 4.2. Thermal-Response Evaluation

**Figure 4:**
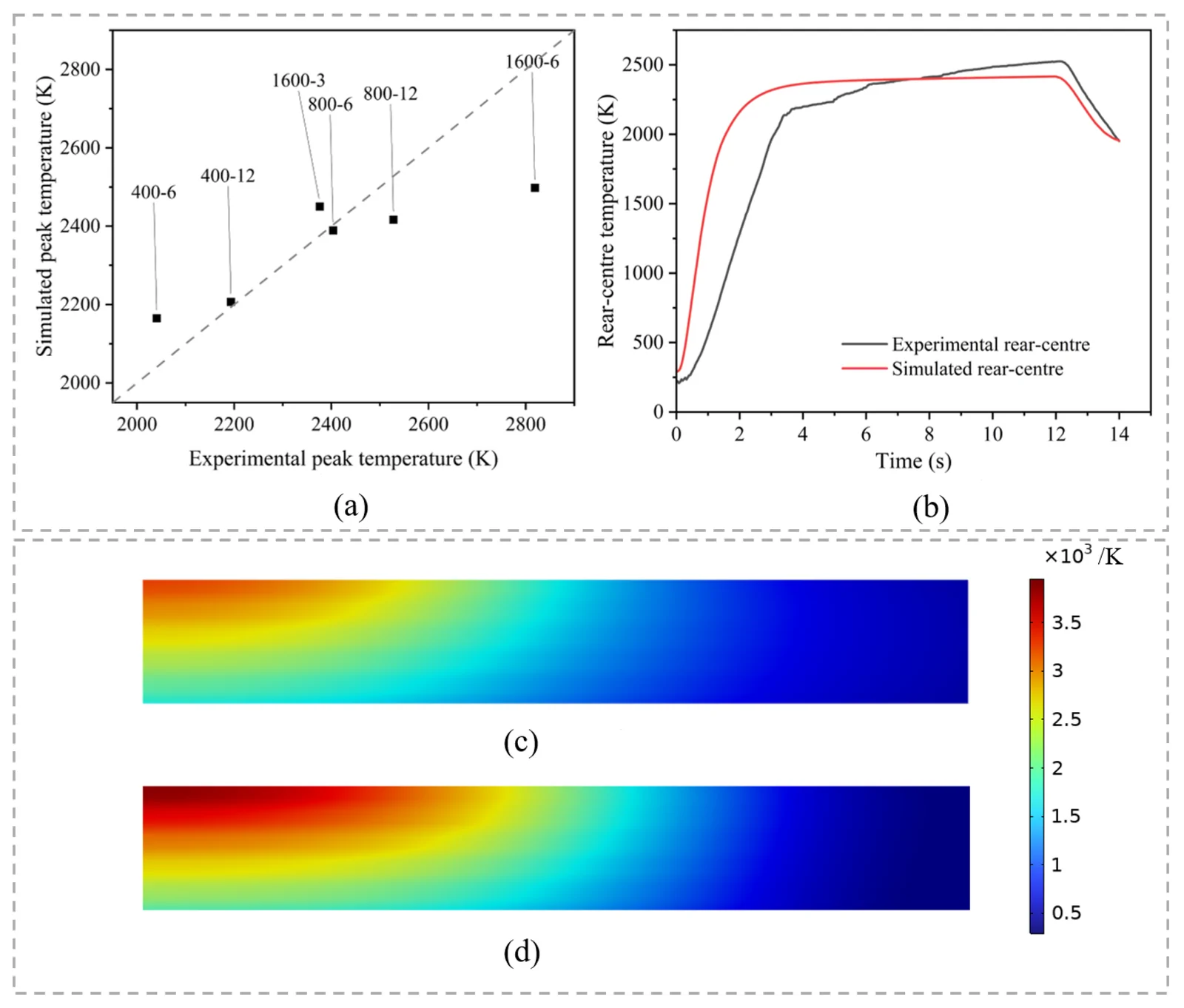
Evaluation of the thermal response: (**a**) comparison of experimental and simulated peak rear-centre temperatures for all six conditions, with the dashed line denoting *y* = *x*; (**b**) experimental and simulated rear-centre temperature histories for the 800–12 condition; (**c**) temperature field at the end of 800 W·cm^−2^ irradiation for 12 s; and (**d**) temperature field at the end of 1600 W·cm^−2^ irradiation for 6 s. Panels (**c**,**d**) share the same temperature scale.

Figure 4a compares experimental and simulated peak rear-centre temperatures for all six conditions, and Figure 4b shows the representative 800–12 history retained in the main text. The peak-temperature mean absolute percentage error was 4.37%. This scalar metric indicates agreement in peak magnitude but does not validate the complete transient response. Complete available histories for all six conditions are provided in Dataset S11 rather than added to the main text, because the condition-dependent heating-stage discrepancies are important but secondary to the phase-state interpretation.

Dataset S11 shows that the calculations generally heat earlier and faster than the measurements, with condition-dependent differences in peak timing and cooling. Four model exports end at laser shut-off, whereas 400–12 and 800–12 also include 6 s of simulated cooling; this coverage is labelled explicitly in the dataset. These discrepancies are not represented by the peak-temperature MAPE and indicate uncertainty in transient heat storage, boundary heat loss and fixed effective properties. The thermal results are therefore described as calibrated response estimates, not independent validation.

The previously measured inner boundary of the surface transition zone provides a secondary reference for the radial thermal field [25]. For 800–12, the experimental and simulated values were 2707.9 and 2810.4 K, respectively (+3.78%); for 800–6, they were 2714.5 and 2999.9 K (+10.5%). The larger short-duration discrepancy indicates excessive early concentration of surface energy in the model and is retained as a limitation of the transient calibration.

Figure 4c,d shows end-of-irradiation temperature fields for 800–12 and 1600–6 on a common scale. In both cases, temperature decays radially and through the thickness from the irradiated centre. The highest-temperature region is more concentrated near the front face under 1600–6 because of rapid energy deposition at high power density, whereas the longer 800–12 exposure allows more heat to penetrate the interior. Changes in gradient across alternating layers reflect the different effective properties of fibre and web layers and establish depth-dependent reaction conditions.

A three-level macroscale mesh study was performed for the representative 800–12 condition. The coarse, medium and fine meshes contained approximately 2.28 × 10^5^, 4.68 × 10^5^ and 7.36 × 10^5^ elements, respectively. From the medium to fine mesh, rear-centre peak temperature, centreline *Y*_SiC_ and centreline *ρ*_rel_ changed by 0.286%, 0.368% and 0.290%, respectively (Dataset S15A). The fine mesh was used for the reported calculations. This test addresses the macroscale transient solution; no new RVE statistical-convergence claim is made.

A bounded one-at-a-time sensitivity analysis was also conducted around the reported 800–12 parameter set (Dataset S15B). The tested SiC kinetic variation changed rear-centre peak temperature by at most 0.12%, centreline *Y*_SiC_ by 4.00%, centreline *ρ_rel_* by 2.73% and maximum retained SiO_2_ density by 3.89%; the tested product-loss variation changed maximum retained SiO_2_ density by up to 16.67% while changing the other reported metrics by less than 0.2%. Thus, the principal qualitative conclusion—separate constituent consumption and product-retention responses—is retained, although local SiO_2_ magnitude is the most parameter-sensitive output.

**Figure 5:**
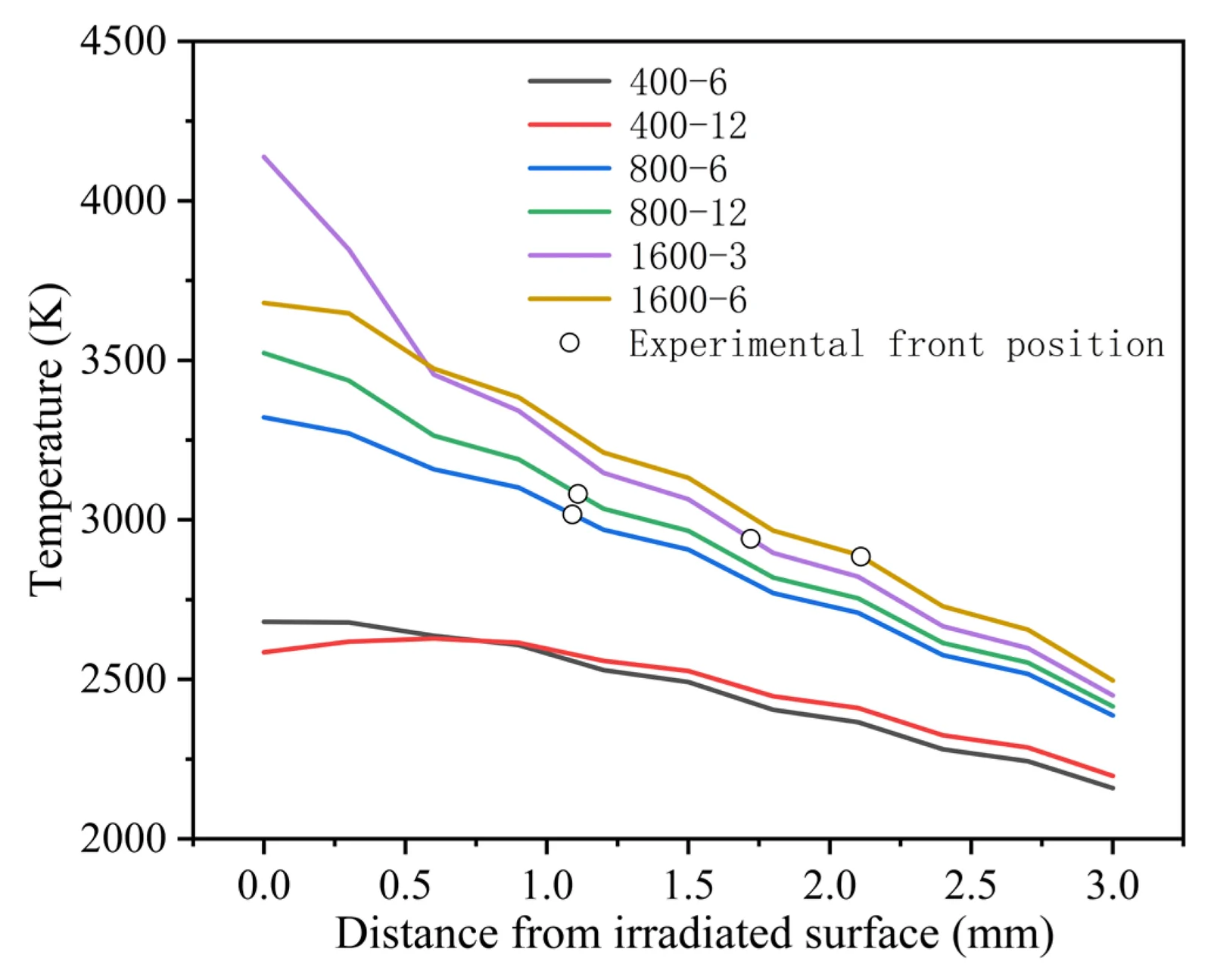
Centreline temperature profiles for the six conditions and positional mapping of the experimental internal degradation fronts. Open symbols denote simulated temperatures sampled at the experimentally measured front depths.

Figure 5 maps each measured front coordinate onto the corresponding simulated centreline temperature profile. The sampled values for 800–6, 800–12, 1600–3 and 1600–6 were 3017.6, 3081.5, 2941.2 and 2885.2 K, respectively. This is an experimental-to-model mapping, not a model-predicted front. The mapped *Y*_SiC_ and *ρ*_rel_ values at the four coordinates did not collapse to a defensible common state threshold; imposing a retrospective threshold would therefore be circular and was not attempted. No marker is shown for the 400 W·cm^−2^ conditions because no resolvable front was observed.

The sampled values suggest that the experimentally visible interfaces occurred within a similar high-temperature region under the investigated conditions. Their spread also indicates that a single temperature does not uniquely define the measured boundary and that exposure duration, phase consumption and experimental detectability contribute to its position. The 2890–3080 K interval is therefore reported only as a condition-specific mapping for the present material and loading cases, not as a universal degradation criterion. The 1600–6 value is additionally subject to the largest geometric uncertainty because the fixed-domain model does not represent the measured 1.21 mm surface recession.

### 4.3. Phase Evolution and Product Retention

**Figure 6:**
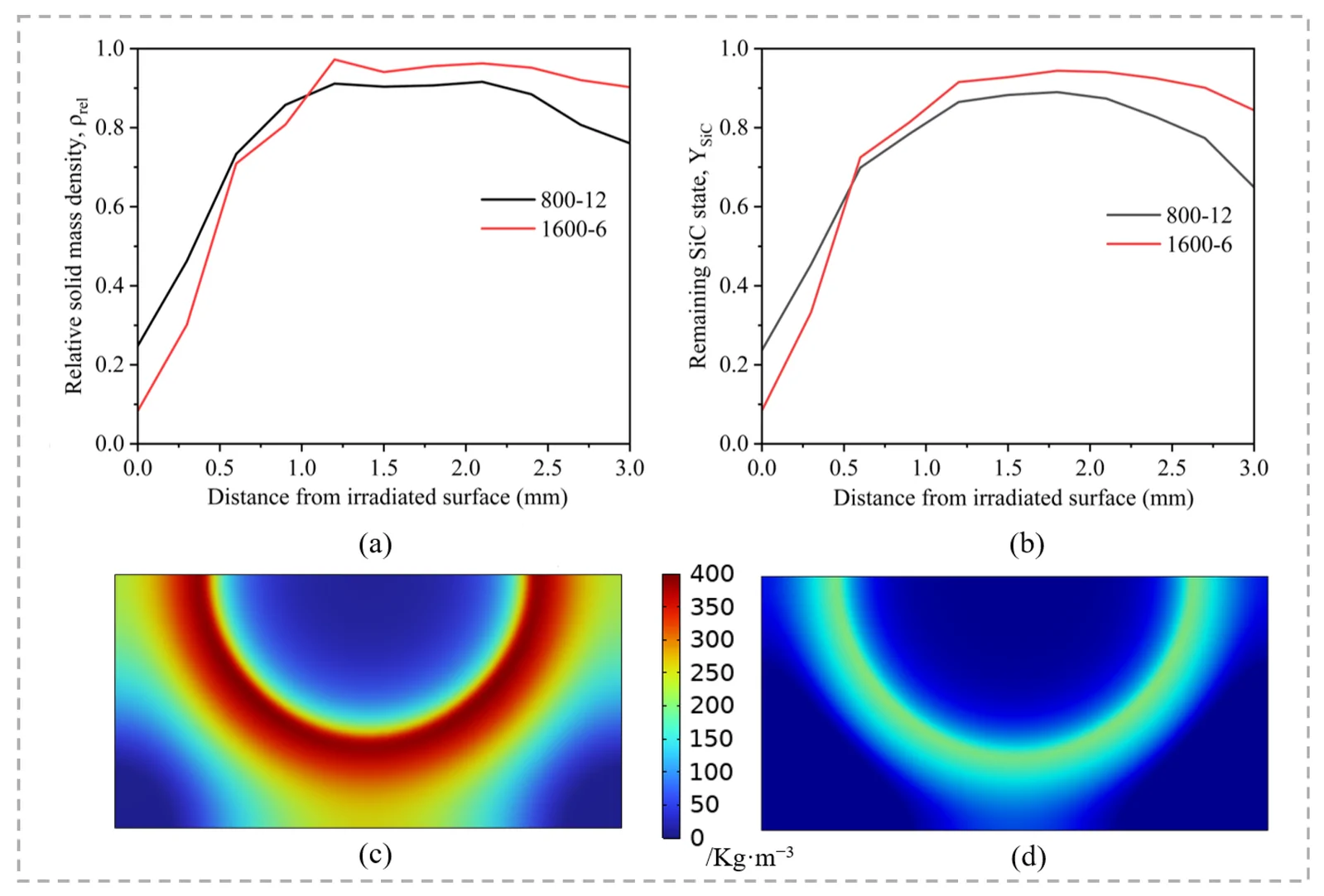
Solid-phase states and near-surface SiO_2_ distributions at the end of irradiation for the 800–12 and 1600–6 conditions: (**a**) centreline relative solid mass density, *ρ*_rel_; (**b**) centreline remaining SiC state, *Y*_SiC_; (**c**) near-surface current SiO_2_ mass density, *ρ*_SiO2,cur_, after irradiation at 800 W·cm^−2^ for 12 s; and (**d**) *ρ*_SiO2,cur_ after irradiation at 1600 W·cm^−2^ for 6 s. Panels (**c**,**d**) share the same colour scale.

After the thermal response was evaluated, the local material state was examined using the remaining SiC state, $Y_{SiC}$, and the relative solid mass density, $\rho_{rel}$, as shown in Figure 6a,b. At the irradiated surface under 800–12, $\rho_{rel}$ and $Y_{SiC}$ decreased to approximately 0.25 and 0.24, respectively, whereas both decreased to approximately 0.08 under 1600–6. The two variables gradually recovered towards their initial values with increasing depth and exhibited local changes near the alternating layer interfaces. Within the model, the lower near-surface values under 1600–6 indicate greater SiC consumption and total solid-mass loss, consistent with the larger measured recession and internal-front depths under this condition.

Although $\rho_{rel}$ and $Y_{SiC}$ follow similar through-thickness trends, they describe different aspects of degradation. $Y_{SiC}$ represents the remaining SiC phase, whereas $\rho_{rel}$ additionally incorporates carbon consumption and mass changes associated with SiO_2_ formation and loss. Their combined interpretation therefore distinguishes SiC-matrix degradation from the total evolution of retained solid mass. The calculated profiles are qualitatively consistent with the low-grey-value CT region and the Si-signal transition observed by EDS, but they are not treated as direct quantitative measurements of local phase composition.

The near-surface current SiO_2_ mass density, $\rho_{SiO_{2},cur}$, describes the competition between oxide formation and subsequent product loss, as shown in Figure 6c,d. The 800–12 condition produced a pronounced annular retention zone with a maximum of approximately 360 kg·m^−3^, whereas the maximum under 1600–6 was approximately 180 kg·m^−3^. Within the model, the reduced SiO_2_ retention at the higher thermal load results from intensified high-temperature product loss in the central region, while the cooler peripheral region remains more favourable for oxide preservation.

Consequently, retained SiO_2_ should not be interpreted as a monotonic indicator of total degradation. The irradiated centre combines strong SiC consumption with limited oxide retention, whereas the surrounding region experiences less severe constituent consumption and retains more SiO_2_. This calculated central-consumption and peripheral-retention pattern is consistent with the experimentally observed surface zonation and supports the distinction between product distribution, total solid-mass loss and internal degradation.

### 4.4. Mechanistic Interpretation of Internal Degradation

Surface recession and internal degradation are experimentally distinguishable but non-coincident manifestations of laser damage. The measured front lies below the final recession surface, whereas the phase-selective model calculates progressive constituent and solid-mass changes within the retained material. Mapping the experimental coordinates to the model places them within a similar simulated high-temperature region, but this correspondence is condition-specific and does not establish a universal reaction threshold or an independently predicted state boundary.

The calculated phase fields help interpret this progression. Lower near-surface YSiC and rhorel under 1600–6 indicate stronger modelled SiC consumption and solid-mass loss than under 800–12. Retained SiO_2_ shows a different calculated trend, with the maximum decreasing from approximately 360 kg per cubic metre under 800–12 to 180 kg per cubic metre under 1600–6. This result suggests increased competition between oxide formation and high-temperature loss at the higher load, while the experimental CT and EDS observations provide qualitative structural and elemental context rather than quantitative phase validation.

Taken together, the observations and calculated fields support an inward degradation sequence in which radial and through-thickness heating activates constituent consumption near the surface, while subsurface material can remain geometrically present after partial thermochemical modification. The pit bottom is therefore treated as a geometric-removal boundary, whereas the deeper CT interface records an experimentally visible state transition. The specific contributions of SiC depletion, carbon consumption and pore development remain a model-based mechanistic interpretation that requires quantitative local density and phase measurements for independent verification.

### 4.5. Assumptions, Uncertainty and Scope

The model solves a normalised continuum oxygen diffusion–reaction field and uses effective reaction kinetics and smooth stock limits. Although this is more physically explicit than a single empirical oxygen multiplier, a_O_2_ is not an absolute oxygen concentration or partial pressure. The formulation does not resolve the external gas boundary layer, crack-scale transport, pressure-dependent passive-to-active transitions or evolving permeability. It therefore provides a semi-quantitative description of oxygen availability and phase evolution rather than unique identification of elementary transport and reaction constants.

The computational domain remains fixed and the initial effective density, conductivity and heat capacity are retained as degradation proceeds. Reaction heat is coupled to the energy equation, but surface recession and degradation-dependent thermophysical properties are not. SiC consumption, pore development and solid-mass loss would be expected to change conductivity and volumetric heat capacity, while recession shortens the remaining conduction path. These competing effects are most important for 1600–6, where the measured recession reached approximately 40% of the original thickness; late-stage fields for this condition are therefore interpreted semi-quantitatively.

The experimental comparison is also limited by two independently irradiated specimens per condition and a reconstructed CT voxel size of 25 μm. Section-to-section measurements quantify interface undulation and measurement repeatability but do not increase the independent sample size. CT grey values were not calibrated to absolute local density, three-dimensional porosity was not segmented, and EDS did not provide quantitative local phase fractions. Consequently, the calculated constituent and density states are compared qualitatively with the observed interface.

The investigated scope comprises one needled 2.5D architecture, one specimen thickness, quiescent air, one laser wavelength and six loading conditions. The mapped temperatures and calculated phase trends should not be transferred directly to other composite architectures, thicknesses, atmospheres or irradiation regimes without new thermal calibration and experimental comparison.

## 5. Conclusions

This study combined reanalysed infrared thermography, micro-CT and SEM/EDS observations with a layered phase-selective degradation model to distinguish surface recession from internal thermochemical degradation in 2.5D C/SiC composites. The main conclusions are as follows:

1. Micro-CT identified a continuous internal degradation front beneath the final recession surface for all four conditions with resolvable damage. The separation between the two boundaries ranged from 0.84 to 1.07 mm, demonstrating that surface recession alone does not represent the full through-thickness extent of laser-induced modification. At 800 W·cm^−2^, the 0.02 mm difference between the mean front depths after 6 and 12 s was below the 25 μm voxel size and within specimen-to-specimen uncertainty and therefore was not interpreted as resolved front advancement.
2. The calibrated layered model matched the magnitude of the rear-centre peak temperatures across the six investigated conditions, with a mean absolute percentage error of 4.37%, although larger discrepancies remained during transient heating and cooling. Temperatures sampled at the experimentally measured front coordinates ranged from approximately 2890 to 3080 K. This range associates the observed interfaces with a high-temperature reaction region under the present conditions, but it represents experimental-to-model field mapping rather than an independently predicted or universal degradation threshold.
3. The calculated phase fields distinguished geometric surface removal from phase degradation within the retained material. Compared with 800–12, the 1600–6 condition produced greater near-surface SiC consumption and solid-mass loss, while the maximum retained SiO_2_ density decreased from approximately 360 to 180 kg·m^−3^ as high-temperature product loss increasingly competed with oxide formation. These results support an interpretation in which the visible pit represents macroscopic material removal, whereas the deeper CT interface is associated with cumulative constituent consumption and pore development. This interpretation remains subject to the fixed-grid, fixed-property and effective-kinetics assumptions of the present model.

## Figures and Tables

**Table 1:** Continuous-wave laser irradiation conditions.

Condition	Power Density (W·cm^−2^)	Irradiation Time (s)
400–6	400	6
400–12	400	12
800–6	800	6
800–12	800	12
1600–3	1600	3
1600–6	1600	6

**Table 2:** Main geometric and heat-transfer parameters of the macroscale layered model.

Parameter	Symbol	Value
Specimen length	L_x_	40 mm
Specimen width	L_y_	40 mm
Specimen thickness	H	3 mm
Symmetry fraction	*f*_sym_	1/2
Total number of layers	N_l_	10
Number of fibre layers	N_f_	5
Number of web layers	N_w_	5
Single-layer thickness	δ_l_	0.3 mm
Fibre-layer orientation	θ_f_	0°/90° alternating
Initial effective density	*ρ*_0_	1940 kg·m^−3^
80% energy radius	*R*_80_	10.5 mm
Energy fraction within R_80_	*f*_80_	0.80
Gaussian 1/e^2^ radius	*w*_0_	11.7049 mm
Effective absorptivity	*η*_abs_	0.72
Initial temperature	*T*_0_	293.15 K
Ambient temperature	*T*_∞_	293.15 K
Front-face convection coefficient	*h*_top_	5 W·m^−2^·K^−1^
Side-face convection coefficient	*h*_side_	5 W·m^−2^·K^−1^
Rear-face convection coefficient	*h*_back_	5 W·m^−2^·K^−1^
Surface emissivity	ε	0.95
Stefan–Boltzmann constant	σ	5.6704 × 10^−8^ W·m^−2^·K^−4^
Through-thickness conductivity factor	f_z_	1.00
Lower interpolation temperature	*T*_min_	293.15 K
Upper interpolation temperature	*T*_max_	3500 K

**Table 3:** Experimental degradation dimensions and comparison of peak rear-centre temperatures for the six conditions.

Condition	Surface Recession (mm)	Front Depth (mm)	Experimental Peak Temperature (K)	Simulated Peak Temperature (K)
400–6	N.R.	N.R.	2040	2164
400–12	N.R.	N.R.	2193	2206
800–6	0.19 ± 0.01	1.09 ± 0.04	2403	2389
800–12	0.27 ± 0.03	1.11 ± 0.03	2527	2416
1600–3	0.65 ± 0.06	1.72 ± 0.07	2376	2450
1600–6	1.21 ± 0.07	2.11 ± 0.07	2819	2498

## Data Availability

The original contributions presented in this study are included in the article/Supplementary Materials. Further inquiries can be directed to the corresponding author.

## Supplementary Materials

The following supporting information can be downloaded at: https://www.mdpi.com/article/10.3390/ma19163558/s1. File S1: Supplementary_Data_Revised.xlsx, containing Tables S1–S9 (RVE hierarchy, phase allocation, temperature-dependent properties, final model settings, oxygen/reaction formulation and numerical guards), Dataset S10 (reanalysed experimental and thermal summary), Dataset S11 (available rear-centre temperature histories for all six conditions), Datasets S12–S14 (centreline temperature, experimental-front mapping and phase-state profiles), and Dataset S15 (macroscale mesh and parameter-sensitivity checks).

## Abbreviations

The following abbreviations are used in this manuscript:

2.5D	two-and-a-half-dimensional
C/SiC	carbon-fibre-reinforced silicon carbide
CC BY 4.0	Creative Commons Attribution 4.0 International
CT	computed tomography
EDS	energy-dispersive X-ray spectroscopy
micro-CT	X-ray micro-computed tomography
PAN	polyacrylonitrile
RVE	representative volume element
SEM	scanning electron microscopy
